# T cells display mitochondria hyperpolarization in human type 1 diabetes

**DOI:** 10.1038/s41598-017-11056-9

**Published:** 2017-09-07

**Authors:** Jing Chen, Anna V. Chernatynskaya, Jian-Wei Li, Matthew R. Kimbrell, Richard J. Cassidy, Daniel J. Perry, Andrew B. Muir, Mark A. Atkinson, Todd M. Brusko, Clayton E. Mathews

**Affiliations:** 1 Department of Pathology, Immunology, and Laboratory Medicine, University of Florida, Gainesville, FL USA; 2 Department of Pediatrics, Emory University, Atlanta, GA USA; 3 Present Address: Department of Endocrinology and Metabolism, West China Hospital of Sichuan University, Chengdu, 610041 China; 4 Present Address: Department of Radiation Oncology, Emory University, Atlanta, GA USA

## Abstract

T lymphocytes constitute a major effector cell population in autoimmune type 1 diabetes. Despite essential functions of mitochondria in regulating activation, proliferation, and apoptosis of T cells, little is known regarding T cell metabolism in the progression of human type 1 diabetes. In this study, we report, using two independent cohorts, that T cells from patients with type 1 diabetes exhibited mitochondrial inner-membrane hyperpolarization (MHP). Increased MHP was a general phenotype observed in T cell subsets irrespective of prior antigen exposure, and was not correlated with HbA1C levels, subject age, or duration of diabetes. Elevated T cell MHP was not detected in subjects with type 2 diabetes. T cell MHP was associated with increased activation-induced IFNγ production, and activation-induced IFNγ was linked to mitochondria-specific ROS production. T cells from subjects with type 1 diabetes also exhibited lower intracellular ATP levels. In conclusion, intrinsic mitochondrial dysfunction observed in type 1 diabetes alters mitochondrial ATP and IFNγ production; the latter is correlated with ROS generation. These changes impact T cell bioenergetics and function.

## Introduction

Increasing evidence suggests that type 1 diabetes patients exhibit immune dysregulation, most notably, a propensity towards pro-inflammatory innate immune activities and aberrant adaptive T cell responses^1^. Despite this apparent deficit in immune tolerance, the cellular and molecular contributors to this process remain poorly characterized. The essential role of mitochondria in T cell activity has drawn great attention in recent years^2, 3^. Metabolic control of adaptive T cell activity likely plays a critical role in determining autoimmune disease progression or the maintenance of peripheral immune tolerance since, in these processes, mitochondrial metabolic activity plays a central role in controlling T cell activation, proliferation, and programmed cell death^4^.

In addition to providing energy for most human cells, mitochondria are also a major site for generation of reactive oxygen species (ROS). When T cells interact with antigen presenting cells (APCs) through HLA/antigen-T cell receptor (TCR) engagement, mitochondria within T cells are translocated to the region of the cytoplasm directly adjacent to the immunological synapse. At the immunological synapse, through a balanced process of fission and fusion, mitochondria maintain inner-membrane potential (ΔΨm), generate ATP, control local calcium concentrations, and produce mitochondrial ROS (mtROS)^5, 6^. This generation of mtROS is essential for IL-2 production and proliferation^7^. Therefore, mitochondria are not only the T cell powerhouse but also, essential for regulating cell signaling. Given these processes are known to play a role in controlling immune tolerance, it is possible that dysfunction of mitochondria could result in immune dysregulation and autoimmunity.

T cell mitochondrial dysfunction has been identified as a feature in multiple autoimmune diseases, including Systemic Lupus Erythematosus (SLE)^8–10^. In human SLE, the phenotype of persistent mitochondrial inner membrane hyperpolarization (MHP) is restricted to T cells. T cell MHP has been associated with elevated cellular ROS levels^11^. Further, increased production of Nitric Oxide (NO) by monocytes is thought to be the mechanism for induction of T cell MHP in SLE patients^12^. In type 1 diabetes, studies linking mitochondrial defects to disease are near exclusively limited to murine models where mitochondrial control of autoimmunity has been linked with dysregulated T cell apoptosis. Indeed, in both diabetes-prone NOD mice and BB-DP rats, genetic susceptibility regulates the expression of genes controlling mitochondrial apoptosis of T cells^13, 14^, resulting in autoimmunity. However, as noted, there is a paucity of studies of mitochondrial function or of metabolic control in T cells in human type 1 diabetes.

In this study, we first analyzed T cell ΔΨm using peripheral blood mononuclear cells (PBMC) from type 1 diabetes patients and controls. We observed that T cells of all subsets from type 1 diabetes patients exhibit MHP, which is not associated with age, disease duration, or metabolic control of the subjects. We then confirmed this observation in enriched total T cells from a separate cohort, which included a group of patients with type 2 diabetes to determine whether T cell MHP is a consequence of abnormal glucose metabolism. Analyses indicated that T cells from patients with type 2 diabetes did not demonstrate T cell MHP. Functional studies provided evidence that T cell MHP was linked with altered mitochondrial and cytokine responses from T cells of patients with type 1 diabetes after TCR stimulation.

## Results

### Low dose DiOC6 is specific for mitochondria

**Figure 1:**
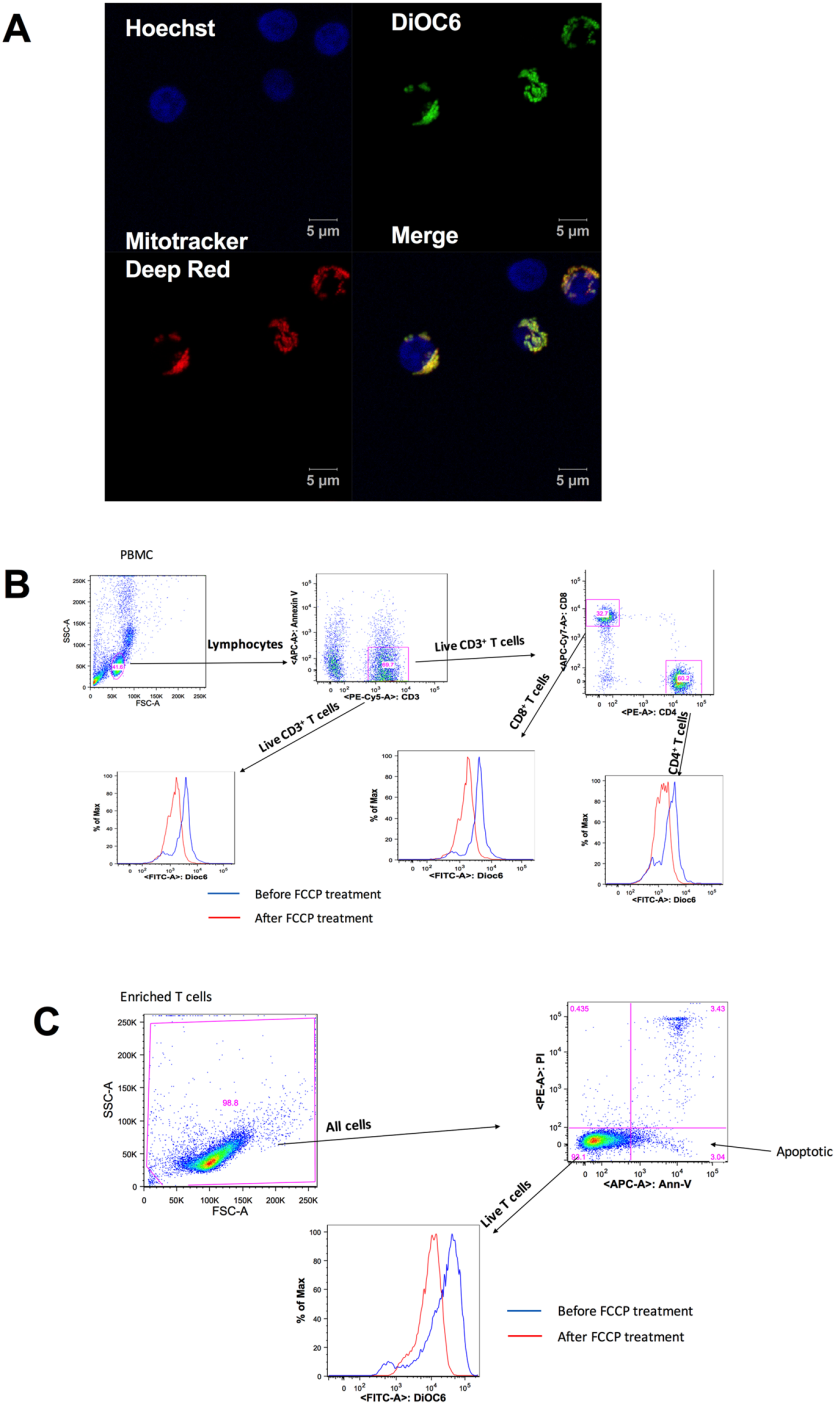
Staining and gating strategies for ΔΨm measurement. (**A**) Confocal image shows co-localization of low dose DiOC6 (20nM) and Mitotracker Deep Red in enriched human T cells. Green: DiOC6; Red: Mitotracker Deep-Red; Blue: Hoechst 33258. (**B**) Gating strategy for measuring T cell ΔΨm in human PBMC. (**C**) Gating strategy for measuring ΔΨm and apoptosis in enriched human T cells. ΔΨm was expressed as MFI of DiOC6 that was corrected with the value from the same sample after FCCP treatment as mentioned in method session.

To rule out the possibility of DiOC6 staining other negatively charged organelles, we performed confocal imaging analysis. At 20nM concentration, DiOC6 overlaps with mitochondrial dye Mitotracker Deep Red (Figs. 1A, S1, Supplemental video 1). Image analysis indicated that low dose DiOC6 and Mitotraker Deep Red co-localize (Table S1). Therefore, DiOC6 was used in subsequent ΔΨm analysis. Figures 1B and C indicate the gating strategy for measuring T cell ΔΨm from PBMC and enriched T cells, respectively. Figure 1C also indicates the analysis of apoptosis by staining with Annexin-V and Propidium Iodide.

### T cells from patients with type 1 diabetes exhibit MHP

**Figure 2:**
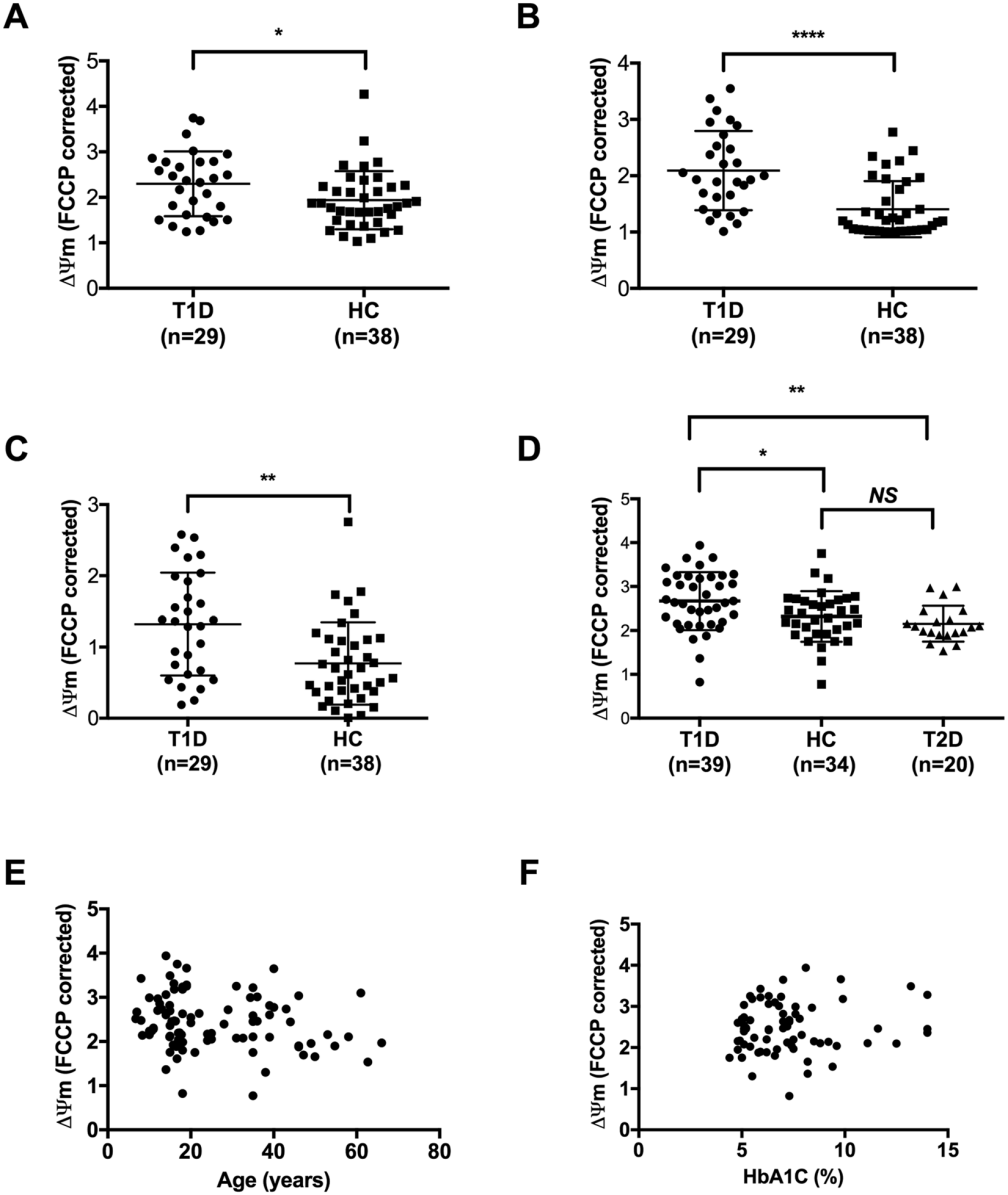
T cells from patients with type 1 diabetes exhibited MHP. T cells from peripheral blood of type 1 diabetes patients (T1D; n = 29) exhibited a higher mitochondrial membrane potential (ΔΨm) when compared to T cells from healthy controls (HC; n = 38) in (**A**) CD4^+^, (**B**) CD8^+^ and (**C**) CD3^+^ subsets; (**D**) In an independent cohort, enriched total T cells from fresh blood of T1D patients (n = 39) showed higher ΔΨm when compared to HC individuals (n = 34) or patients with type 2 diabetes (n = 20). T cell ΔΨm was not correlated with (**C**) age or (**D**) HbA1c level. *P < 0.05, **P < 0.005, ****P < 0.001. Mann-Whitney test.

To study T cell mitochondrial function, we first analyzed ΔΨm in T cells from fresh PBMC by flow cytometric analysis. When compared to healthy controls, the T cells of patients with type 1 diabetes exhibited significant MHP (Fig. 2A–C). In individuals with T cell MHP, the phenotype was detected in CD4^+^ (Fig. 2A; *P* < 0.05), CD8^+^ (Fig. 2B; *P* < 0.0001), and total CD3^+^ (Fig. 2C; *P* = 0.001) T cell populations. These data suggested that MHP was a general characteristic of T cells in patients with type 1 diabetes.

To determine if T cell MHP was associated with activation or the transition from a naïve to differentiated T cell phenotype, we initiated a second phase of the study and recruited an additional 107 participants (Table 1). We analyzed ΔΨm in each subset of T cells by co-staining PBMC with markers to identify naïve and memory T cell compartments as well as markers of T cell activation status. A total of 10 groups of lymphocytes, including 9 subsets of T cells were defined. Individuals with T cell MHP exhibited this phenotype globally across naïve (CD45RA^+^ CD45RO^−^) and memory (CD45RO^+^CD45RA^−^) subgroups of both CD4^+^ and CD8^+^ T cells, as well as conventional (CD127^+^CD25^−^) and regulatory (CD127^−^CD25^+^) CD4^+^ T cell subsets. Pairwise correlation analysis failed to detect strong associations between the ΔΨm and any of the nine T cell subsets analyzed (See Supplementary Table S1). Further, T cell MHP was not associated with any change in resting viability, altogether indicating that when MHP was present, it was consistently observed in all T cell subsets regardless of their activation status. In sum, MHP appeared to be a phenotype that was intrinsic to all T cell subsets.

**Table 1:** Research Subjects Participating in the Three Study Phases.

Phase	Participant Group	n	Age [Years(Medianrange)]	Gender [% Female]	Disease Duration [Years (Median, range)]	HbA1C [%]
1		67				
	Type 1 diabetes	29	14, 9–27	55%	5.6, 0.1–18.6	7.1–14.6
	Healthy control	38	16, 7–38	42%	N/A	ND
2		107				
	Type 1 diabetes	25	12, 4–60	44%	2.67, 0.08–18	ND
	Healthy Control	21	33.5, 19–51	52%	N/A	ND
	Type 2 diabetes	1	45	100%		ND
	1st Degree Relative	53	30, 4–56	60%		
	2nd Degree Relative	7	60, 50–66	86%		
3		93				
	Type 1 diabetes	39	18, 6.8–61	51%	8, 0.08–35.1	9.9–14
	Healthy control	34	22.5, 7–53	62%		ND
	Type 2 diabetes	20	34.5, 10.6–66	65%	3.25, 0.08–22	4.8–14
Functional Studies		137				
	All Phase 3	93				
	Healthy control	3	44, 22–45	67%		ND
	1st Degree Relative	40	32.5, 8–61	48%		ND
	Type 2 diabetes	1		0%		ND

**Figure 3:**
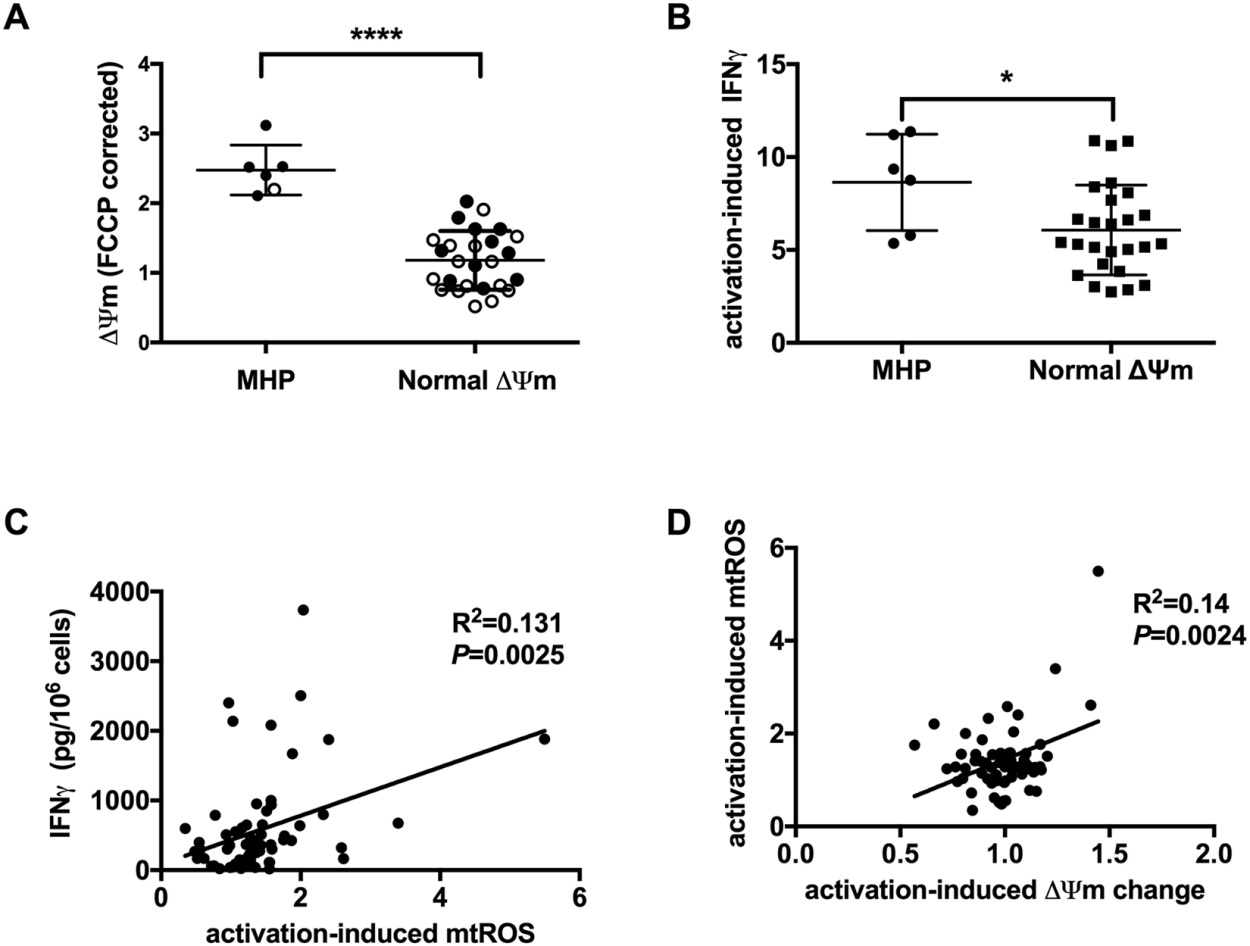
T cell MHP is associated with functional changes upon activation. (**A**) T cell ΔΨm of individuals whose cryopreserved PBMC samples were selected for *in vitro* activation. Black dot: type 1 diabetes patients. Open circles: healthy controls. (**B**) CD4^+^ T cells from cryopreserved PBMC of individuals with MHP (n = 6, including 5 type 1 diabetes patients and 1 healthy control) show higher intracellular IFNγ staining after 72-hour stimulation with plate-bound anti-CD3 and anti-CD28, when compare to cells from individuals without MHP (n = 26, including 10 type 1 diabetes patients and 16 healthy controls). (**C**) Mitochondria-specific ROS produced by enriched total T cells after stimulating by plate-bound anti-CD3 and anti-CD28 for 24 hours, is correlated with the amount of IFNγ secreted in the supernatant. (**D**) Activation-induced change of ΔΨm is positively correlated with mt-specific ROS production after 24-hour stimulation *in vitro*. *P < 0.05, ****P < 0.0001, Mann-Whitney test (**A** and **B**). Linear regression (**C** and **D**).

Given that MHP in subsets of subjects was consistent among all T cell subsets regardless of their activation status, we further analyzed ΔΨm using enriched total T cells in an independent cohort of research participants (Phase 3; Table 1). We were able to confirm the link between T cell MHP and type 1 diabetes (Fig. 2D; *P* = 0.018) identified in phases 1 of this study. To further analyze the effect of hyperglycemia on T cell ΔΨm, a group of patients with type 2 diabetes (n = 20) was included in this cohort (Phase 3; Table 1). The HbA1c level of the type 1 diabetes group was 7.9 ± 2.1 (%, Mean ± SD) while that of the type 2 diabetes group was 7.8 ± 2.6. T cell ΔΨm from type 2 diabetes patients was indistinguishable from controls (Fig. 2D) and significantly lower than the type 1 diabetes group (*P* = 0.0005). When analyzing all three phases of the study, no correlations were detected between: (1) ΔΨm and age (Fig. 2E), (2) ΔΨm and HbA1c levels (Fig. 2F), (3) ΔΨm and duration of diabetes (See Supplementary Fig. S2A), or (4) ΔΨm and glucose levels at blood sampling (See Supplementary Fig. S2B). Together, these data suggest that MHP is intrinsic to T cells from patients with type 1 diabetes and is not a consequence of abnormal glucose control.

### MHP is associated with functional changes in T cells after activation

Cryopreserved PBMC samples from Phase 1 individuals (Table 1 and Fig. 1A–C) were chosen, including a total of 6 with MHP (5 type 1 diabetes, 1 control) and 26 with normal ΔΨm (10 type 1 diabetes and 16 controls; Fig. 3A) to determine the effect of MHP on T cell function. Cells were stimulated *in vitro* with plate-bound anti-CD3, soluble anti-CD28 for 72 hours. Using Cell Trace Violet, we observed that cell proliferation was not significantly different when comparing MHP versus normal ΔΨm groups (*P* = 0.145). However, intracellular IFNγ staining indicated that CD4^+^ T cells with MHP exhibited elevated IFNγ production when compared to T cells without MHP (Fig. 3B; *P* = 0.02). These data suggest an altered pro-inflammatory T cell effector response is associated with MHP.

T cell mitochondria respond to TCR stimulation by increasing mass, ΔΨm, and ROS generation^15^, and mitochondrial ROS is essential for signal transduction during T cell activation^7, 16^. To study if abnormal mitochondrial responses to T cell activation are linked to T cell dysfunction in type 1 diabetes, total T cells were enriched from fresh peripheral blood and stimulated *in vitro* with plate-bound anti-CD3 and anti-CD28 for 24 hours. Activation-induced IFNγ, detected in the supernatant of anti-CD3/anti-CD28 stimulated cells, was positively correlated with mtROS production (Fig. 3C). Although we did not observe an increase of activation-induced ΔΨm in all T cell samples, the increase in ΔΨm correlated with mtROS production (Fig. 3D). These data linked the type 1 diabetes-associated altered activation-induced mitochondrial response with altered effector T cell cytokine production.

### Lower basal cellular ATP levels observed in T cells of type 1 diabetes subjects

**Figure 4:**
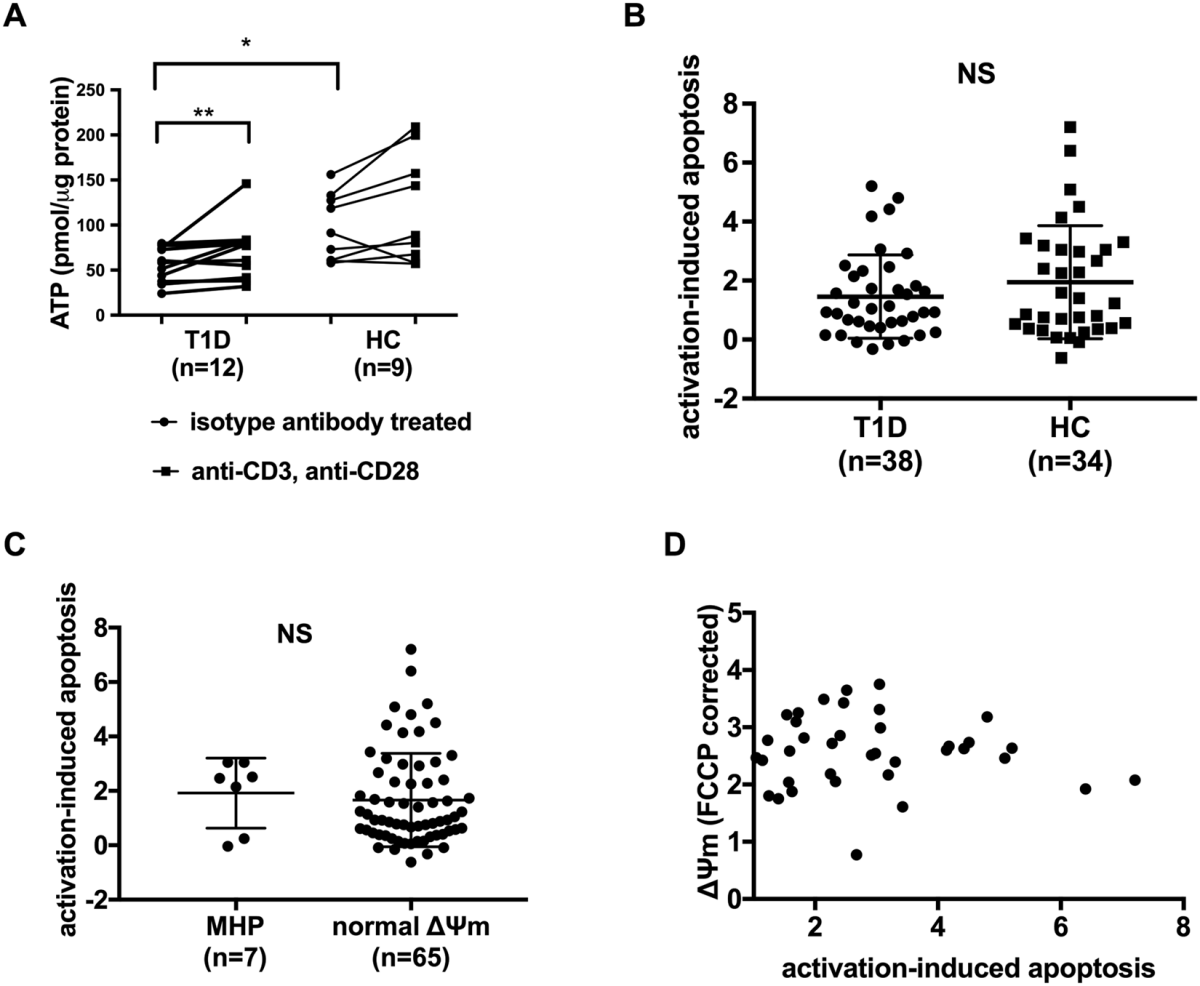
Activation-induced ATP and cell death. (**A**) T cells from type 1 diabetes patients (T﻿1D) have lower basal cellular ATP content compare to healthy controls (HC) but cellular ATP ﻿was﻿ significantly increased after *in vitro* activation. Enriched total T cells were stimulated *in vitro* with plate-bound anti-CD3 and anti-CD28 or isotype antibodies for 24 hours. ATP content was detected in cell lysate and corrected by protein amount. The trend remains that T cells from type 1 diabetes patients have lower ATP content even though *in vitro* activation induces a significant increase of ATP in this group. (**B**) T cells from T1D patients and HC showed the same level of activation-induced apoptosis. After 24-hour activation with plate-bound anti-CD3 and anti-CD28, T cell apoptosis was identified by staining with Annexin-V and Propidium Iodide. Activation-induced apoptosis was calculated as mentioned in method session. (**C**) T cells with MHP showed the same activation-induced apoptosis as normal ΔΨm T cells. (**D**) Activation-induced apoptosis was not correlated with T cell ΔΨm. *P < 0.05, unpaired t test with Welch’s correction. **P < 0.01 Wilcoxon matched pair test for (**A**) and Mann-Whitney test for (**B**).

A major function of mitochondria is the generation of cellular ATP. Although the traditional concept holds that upon activation, T cells utilize glycolysis for energy production (Warburg effect^17^), ATP from mitochondrial oxidative phosphorylation (OXPHOS) is also required during T cell activation and differentiation^18^. Mitochondrial ATP is thought to be important for events that initiate T cell activation. We therefore monitored T cell ATP content after polyclonal stimulation with activating anti-CD3 and anti-CD28 antibodies and used isotype antibody-treated samples to assess basal ATP production. When compared to healthy controls, T cells purified from subjects with type 1 diabetes exhibited significantly reduced cellular ATP content prior to activation (Fig. 4A; *P* = 0.023) suggesting compromised mitochondrial function of these cells. Activation *in vitro* for 24 hours led to an increase of ATP in cells from both patient and control samples. This was not surprising since aerobic glycolysis is the main metabolic pathway after T cell activation^19^. During activation, increased ATP production via glycolysis may be able to facilitate activation-induced signal transduction and downstream cytokine production.

NO is a regulator of mitochondrial biogenesis^20^. At low concentrations, this molecule can inhibit mitochondrial complex IV by competing for oxygen. At higher concentrations, NO can damage electron transport complexes and members of the TCA cycle^21^. NO has been found to be the essential cause of T cell mitochondrial dysfunction in SLE patients^12^. We analyzed T cell NO production 24 hours after polyclonal activation *in vitro* but did not detect significant differences between type 1 diabetes and controls in activation-induced NO production (See Supplementary Fig. S3). Lack of differences in NO production from T cells in type 1 diabetes and controls suggests that, unlike in the case of SLE, the mitochondrial dysfunction in type 1 diabetes is not linked to elevated NO production.

### T cell MHP does not change activation-induced T cell apoptosis

There was no difference in T cell activation-induced cell death/apoptosis (AICD) between type 1 diabetes and control subjects (Fig. 4B ***)***. Similarly, when segregated into MHP group (n = 7: 5 type 1 diabetes, 2 controls) and normal ΔΨm group (n = 65: 33 type 1 diabetes and 32 controls), there was no difference in T cell AICD (Fig. 4C). AICD was not correlated with ΔΨm value (Fig. 4D), nor were changes detected in activation-induced necrosis.

### T cell MHP is not associated with changes in glycolysis

Samples from a subset of participants (n = 29, including 11 patients with type 1 diabetes, 4 healthy controls, 13 relatives, and a patient with type 2 diabetes) in phase 2 were subjected to glycolysis detection with an Seahorse XF24 extracellular flux analyzer. No correlation was detected between T cell MHP and glycolysis (Fig. S4).

## Discussion

Mitochondrial metabolic activity plays a central role in regulating T cell activation, proliferation, and programmed cell death^4^. During T cell activation, mitochondria are recruited to under the immunological synapse where they participate in TCR signaling and T cell proliferation by regulating ATP synthesis, local calcium concentrations^5, 22, 23^, and generate ROS^7, 16^. The roles of metabolic control and mitochondria activity in T cell function have drawn great attention in recent years. Metabolic control of T cell activity may play a critical role in determining autoimmune disease progression through maintenance of peripheral immune tolerance^18^.

In this study, we observed for the first time that T cells from patients with type 1 diabetes exhibit MHP. When MHP was present it was detected in all subsets of T cells regardless of activation status and was not correlated with disease duration or HbA1c level. In addition, MHP was not observed in T cells from patients with type 2 diabetes, whose metabolic status was comparable to the type 1 diabetes group based on HbA1c levels. These data suggest that MHP is an intrinsic T cell defect rather than a consequence of disease status, metabolic control, or recent antigen exposure and activation state. Although DiOC6 can stain other negatively charged membranous structures inside of cells, such as the endoplasmic reticulum, at lower concentrations DiOC6 is specific to mitochondria^24, 25^. In this study, we used a low DiOC6 concentration (20 nM as previously published^15, 26^) to assess ΔΨm in lymphocytes. In addition, using confocal images, we demonstrate that 20nM DiOC6 in combination with the mitochondrial-specific dye Mitotracker-Deep Red are co-localized in both BetaLox 5 cells, with clear mitochondrial structure, and enriched human T cells (Fig. 1A, Supplemental Figure S1, Supplemented video S1, and Table S2 (Co-localization analysis).

Upon TCR stimulation, signaling pathways lead to activation of transcription factors and the production of inflammatory cytokines^27, 28^. The T_H_1 lineage, characterized by high levels of IFNγ production, is considered an important component of type 1 diabetes pathogenesis^29^. Our finding that upon TCR stimulation, CD4^+^ T cells with MHP secrete greater amounts of IFNγ suggests that MHP is linked to differentiation of CD4^+^ T cells toward the T_H_1 lineage^30^. While ROS and cytokines participate in the pathogenesis of type 1 diabetes^31–33^, a recent study using animal models has shown that mitochondria metabolism and ROS production from mitochondrial complex III is required for antigen-specific T cell activation^7^. Moreover, increasing mitochondrial production of hydrogen peroxide by overexpressing superoxide dismutase 2 (SOD2 or MnSOD) has been shown to enhance TCR signaling. In our study, we evaluated mtROS induction by TCR stimulation in combination with co-stimulation. Using enriched total T cells, after a 24-hour stimulation with plate-bound anti-CD3 and anti-CD28, we observed that activation-induced mtROS generation was correlated with IFNγ production. Hence, we hypothesize that dysfunctional mitochondria generate more ROS and further, that increased levels of ROS can stimulate transcription factors which facilitate production of inflammatory cytokines^6^.

T cell MHP has been previously observed in human autoimmunity: in SLE patients, the presence of MHP was linked to lower ATP production and a switch of T cell death from apoptosis to necrosis^26^. Our observation that T cells from type 1 diabetes patients show lower cellular ATP content in a quiescent state is consistent with T cells from SLE being ATP-depleted. However, while in SLE patients, T cell MHP is linked to decreased T cell viability due to elevated T cell necrosis^8–10^, we did not observe any changes of T cell necrosis or apoptosis in type 1 diabetes patients or individuals with T cell MHP.

An additional difference between T cell MHP in type 1 diabetes and SLE is the participation of NO in SLE. In SLE, T cell MHP is highly correlated with NO levels, while in type 1 diabetes we did not observe a link between MHP and NO. NO is a regulator of mitochondrial biogenesis and function. At low concentrations, NO reversibly inhibits mitochondrial cytochrome c oxidase (OXPHOS Complex IV) by competing with oxygen at the active site of the enzyme^34^. At high concentrations, NO can react with superoxide to produce peroxynitrite (ONOO^−^), which can react with and inactivate multiple mitochondrial complexes (I, II, III, IV), the ATP synthetase (ATPase), creatine kinase, as well as aconitase. Further, ONOO^−^ stimulates proton leak through the mitochondrial inner membrane^35^. NO may also facilitate generation of Nitrosothiols (RSNO) that can also inhibit mitochondrial complex I^35^. NO has been found to be the essential cause of T cell mitochondrial dysfunction in human SLE^12^. We did not observe an increase of NO in T cells from type 1 diabetes patients after activation. The difference between the previous observations in SLE patients and our study may be related to the observation that the increased NO in SLE patients originated from monocytes as opposed to T cells, although the same group also reported that NO produced from T cells is increased after activation^12^.

Under normal conditions, TCR stimulation induces a transient decrease of mitochondrial membrane potential within 30 seconds, which is synchronized with a spike in the ATP concentration^36^ that is required for mediating signaling events. Activated T cells then experience a reversible mitochondrial hyperpolarization^10^. That is accompanied by an induction of glycolysis for energy production (Warburg effect^17^) as well as activation of the pentose phosphate pathway (PPP)^37^ for biosynthesis. PPP is regulated by a key enzyme transaldolase^38^. In addition to producing ribose-5 phosphate for nucleic acid synthesis and cell proliferation, PPP also generates NADPH from NADH by the enzyme G6PD and 6-phosphogluconate dehydrogenase (PGD). NADPH is an important reducing agent that reduces glutathione^37, 39^. These changes in PPP are accompanied the reversible MHP in activated T cells. In autoimmune settings like SLE^10^ and type 1 diabetes, the T cell MHP is constant, and our data indicate that in type 1 diabetes, MHP is not restricted to activated T cell subsets only. Although we have not yet examined PPP in these T cells, the persistent MHP in T cells of type 1 diabetes is not likely the result of a PPP associated increase of NADPH and glutathione reduction. On the other hand, the fact that T cell MHP is not associated with glycolytic changes in these cells (Fig. 4S) further suggests that MHP in type 1 diabetes is distinct from activation-induced reversible MHP found in T cells from controls^10^. Our data suggest that the original cause of T cell MHP in type 1 diabetes patients is different from that observed in SLE patients. It is more likely that MHP observed in the T cells of patients with type 1 diabetes results from an intrinsic abnormality of the mitochondria that may arise from the genetic predisposition that is linked to T cell dysregulation in type 1 diabetes.

Indeed, T cell MHP is a novel finding in human type 1 diabetes. MHP is associated with altered T cell response following TCR ligation and co-stimulation. Dysregulated mitochondria in T cells could play a role in disturbances in both central and peripheral mechanisms of immune tolerance. These data demonstrate that altered lymphocyte mitochondrial function and metabolism within type 1 diabetes represent a significant contributor to autoimmune pathogenesis and may constitute a novel therapeutic target for restoring immune regulation. It is worth noting that, although our study indicates that MHP is general to all T cell subsets in type 1 diabetes, it does not indicate that all of these T cell subsets have the same metabolic program. Indeed, it has been well known that different T cell subsets have distinct metabolism characteristics. Future investigations are necessary to study how each T cell subset metabolism changes affect immune balance in the context of type 1 diabetes.

## Methods

### Research subjects

All participants in this three-stage study were enrolled from University of Florida Diabetes Institute clinical network. In the first phase of the study, 38 healthy controls and 29 patients with type 1 diabetes were recruited (Table 1). An extended second-phase study was also conducted to further characterize the activation status of T cells with MHP by enrolling 107 individuals (Table 1). The third phase of this study enrolled 93 individuals to confirm our original observation of MHP as a characteristic of T cells from patients with type 1 diabetes. Samples of all individuals from phase 3 and an additional 44 were proceeded for functional studies (Table 1).

### Study Approval

All studies described within were consented in accordance with an approved study reviewed by the Institutional Review Board of the University of Florida. Written informed consent was received from each participant prior to inclusion in the study.

### Sample preparation

Peripheral blood samples were collected in heparin containing vacutainer tubes (BD Biosciences). For the assays in phase 1 and phase 2, PBMC were isolated within 24 hours of sample collection by first dilute whole blood with 1X D-PBS (no glucose) at 1:1 ratio followed by ficoll gradient^40^. These PBMCs were stained and analyzed by flow cytometry. For the phase 3 immunophenotyping and functional studies, total T cells were enriched using a RosetteSep T cell enrichment kit (Stemcell Technologies).

### T cell ΔΨm analysis

In the first-phase of the study, fresh PBMC were stained with the T cell surface markers anti-CD3 (Clone UCHT1, BioLegend, #300409), anti-CD4 (Clone RPA-T4, BioLegend, #300507), and anti-CD8 (Clone HIT8a, Biolegend, #300925) for 30 minutes at 4 °C, washed, and then stained for ΔΨm with 3,3’-Dihexyloxacarbocyanine Iodide (DiOC_6_; 20nM), a cell-permeable, green-fluorescent, lipophilic dye for 15 minutes at 37 °C. Cells were then washed, Annexin-V (Invitrogen) added, and analyzed using a BD LSR Fortessa flow cytometer. Dead cells were excluded by gating out the Annexin-V positive subset. This 5-color panel was used to detect T cells ΔΨm. ΔΨm was expressed as Mean Fluorescence Intensity (MFI) of DiOC_6_ for each gated T cell subset. To diminish instrumental bias, baseline ΔΨm was obtained by treating each sample with Carbonyl cyanide-4- (trifluoromethoxy) phenylhydrazone (FCCP, 1µM), a mitochondrial uncoupler, for 10 minutes, and the baseline MFI was used to correct for the corresponding DiOC_6_ reading. In the second phase of the study, fresh PBMC were stained with a 9-color panel including the T cell surface markers anti-CD3 (Clone UCHT1, Biolegend, #300424), anti-CD4 (Clone RPA-T4, BD Biosciences, #558116), and anti-CD8 (Clone SK1, Biolegend. #344714), plus the activation markers CD45RA (Clone HI100, BD Biosciences, #560675), CD45RO (Clone UCHL1, Biolegend, #304222), CD127 (Clone HIL-7R-M21, BD Biosciences, 557938) and CD25 (Clone BC96, Biolegend, #302610). Fluorescence minus one (FMO) control was setup for CD25 gating accuracy. Dead cells were gated out using LIVE/DEAD® Fixable Yellow Dead Cell Stain Kit (Invitrogen). ΔΨm was detected as described above. All the above steps were performed using HBSS with no addition of glucose. For the second phase, the cytometer instrumental settings were adjusted to work with the 9-color panel; therefore, the results of this phase were not combined with results from phase 1. Instead, they were analyzed independently.

In the third phase of the study, enriched total T cells were stained for ΔΨm using DiOC_6_ in phenol red-free complete RPMI (Glucose concentration 2 G/L) for 15 minutes at 37 °C, washed with and re-suspended in Annexin-V binding buffer (10 mM HEPES, 140 mM NaCl, 2.5 mM CaCl_2_, 2% FBS, no glucose, PH 7.2). Annexin-V and PI were added before running on flow cytometer. To equalize the effect of environment on mitochondrial function, in each phase of the study, samples from controls and patients were prepared the same way, including reagents concentration, staining time, incubation temperature and duration, washing process, speed, temperature, as well as force and duration of centrifugation. Flow cytometer data were analyzed using Flowjo software (Tree Star Inc. Version 8.8.7-9.7.7).

### T cell activation *in vitro* (72-hour stimulation)

Samples with MHP (ΔΨm greater than median + 1SD) and normal ΔΨm (ΔΨm lower than median + 1SD) were selected from Phases 1 and 3. Twenty-four well plates were coated with crosslinking anti-mouse IgG Fab2 (Jackson Immuno Research) at a final concentration of 1.25 µg/mL in sterile PBS at 4 °C for 16 hours. Crosslinking Fab2 was removed, and then wells were rinsed once with sterile PBS. Either anti-CD3 (OKT3, Biolegend, #317315) or isotype control antibody (IgG2a k isotype ctrl, Biolegend, #40022477) was added at final concentration 1.25 µg/mL and plates were incubated at 37 °C for 3 hours. Coating antibodies were removed and wells rinsed with PBS once before adding PBMC.

Frozen PBMC samples were selected from individuals that participated in phase 1 and included individuals with MHP (n = 6) and those without MHP (n = 26). Samples were thawed, loaded with CellTrace Violet (Life Technologies), and seeded in coated plates at a density of 10^6^ cells per well in 1mL complete RPMI. Soluble anti-CD28 (BD Pharmingen, #555725) or isotype control IgG1 k antibody (ebioscience, #16-4714-85) was added at final concentration 2.5 µg/mL. Cells were stimulated for 72 hours. Golgistop was added for the final 4 hours. After stimulation, cells were stained with LIVE/DEAD™ Fixable Yellow Dead Cell Stain Kit (Life Technologies Ref# L34959), then stained for T cell surface markers, anti-CD3 (Clone OKT3, Biolegend #317318), anti-CD4 (Clone RPA-T4, BD Pharmingen #555347), anti-CD8 (Clone B9.11, Beckman Coulter #IM0451U). Subsequently, cells were fixed and permeablized with BD Cytofix/Cytoperm Kit (BD Biosciences), and stained for intracellular IFNγ (Biolegend #502528). Samples were analyzed by flow cytometry. Dead cells were gated out using Live-Dead yellow positivity. Cell proliferation was analyzed by dilution of CellTrace Violet using Flowjo software to calculate number of peaks, percent divided, proliferation and division indices. T cell IFNγ MFI was assessed under activating conditions (MFI_Act_) as well as in samples treated with isotype antibodies (MFI_Iso_), and activation-induced IFNγ was calculated as (MFI_Act_-MFI_Iso_)/MFI_Iso_.

### T cell activation-induced mitochondrial functional changes (T cells were activated for 24 hours *in vitro* for this purpose)

Total T cells were enriched from fresh peripheral blood using the RosetteSep Human T Cell Enrichment Cocktail (Stemcell Technologies, #15021). Twelve-well plates were coated with anti-CD3 (Biolegend, #317315, clone OKT3, final concentration 10 μg/ml in PBS) or mouse IgG2a, k isotype control (BioLengend, #400224, clone MOPC-173, final concentration 10 μg/ml in PBS) for 3 hours at 37 °C. Coated wells were then rinsed once with PBS and enriched T cells were seeded at 1 × 10^6^ cells/well. Anti-CD28 (BD Biosciences, t#555725, final concentration 20 μg/ml) was added to anti-CD3 coated wells, and mouse IgG k isotype control (eBiosciences, #16-4714-85, final concentration 20 μg/ml) was added to isotype-antibody coated wells. Cells were incubated at 37 °C for 24 hours. After activation, T cell ΔΨm was analyzed by staining with the fluorescent probe DiOC_6_ and corrected with FCCP as mentioned above. mtROS production was detected by staining with the fluorescent mitochondrial specific ROS indicator probe MitoSox-Red (Life techonologies, #M36008, 5 μM) at 37 °C for 15 minutes. Cellular nitric oxide (NO) production was detected by staining with DAF-FM Diacetate (Life techonologies, #D23844, final concentration 2 μM) at 37 °C for 15 minutes. Dead cells and apoptotic cells were excluded by staining with the combination of Annexin-V and PI or 7AAD (BD Biosciences). Data were recorded using a BD LSR Fortessa cytometer. Cellular ATP contents were detected using ATP determination Kit (Life Technologies, # 1577278), read on a BioTek Synergy 2 Multi-Mode Reader, and corrected for protein content measured using the BCA assay (Pierce). Cytokines in culture supernatants were determined using ELISA according to manufacturer recommendations (BD Biosciences). Activation-induced mtROS was calculated as MFI_Act_/MFI_Iso_ of MitoSox-Red staining. Activation induced ΔΨm change was calculated as the ratio MFI_Act_/MFI_Iso_ of DiOC_6_ staining. Activation induced NO production was calculated as MFI_Act_/MFI_Iso_ of DAF-FM Diacetate staining. Activation-induced apoptosis was calculated as the difference in percent Annexin-V positive PI negative apoptotic cells (%Apo) between activating antibody and isotype antibody treated conditions, divided by that of the isotype treated sample [(%Apo_Act_ − %Apo_Iso_)/%Apo_Iso_]. Necrotic cells were gated on PI-positive population (%Necro) and activation-induced necrosis was calculated as [(%Necro_Act_ − %Necro_Iso_)/%Necro_Iso_].

### Glycolysis measurement

Enriched T cells were seeded in a 24-well XF Extracellular Flux Analyzer plate at 5 × 10^5^cells/well in triplicates. Real-time extracellular acidification rates (ECAR) were recorded using an XF Extracellular Flux Analyzer (Fig. S4A). Activating antibodies or respiratory inhibitors were injected at time points as shown in Figure S4A. (a) AntiCD3 (2 µg/mL) plus antiCD28 (1 µg/mL), or isotype antibodies at the same concentrations, (b) ATP synthase inhibitor Oligomycin (12.6 µM), (c) uncoupler Carbonyl cyanide 4-(trifluoromethoxy) phenylhydrazone (FCCP, 1 mM), (d) glycolysis inhibitor 2-DG (44.6 mM), mitochondrial complex I inhibitor Rotenone (1 mM) and mitochondrial complex III inhibitor antimycin A (1 mM). All the above are final concentrations.

### Confocal Microscopy

Human beta cell line BetaLox5 cells^41, 42^ were seeded in chambered glass 1 day prior to staining and imaging. Enriched T cells were stained in Eppendorf tubes before seeding in chambered glass. Cells were stained with DiOC6 20 nM, MitoTracker™ Deep Red FM (Thermo Fisher, M22426) 10 nM, and Hoechst 33258 2 µg/mL for 20minutes at 37 °C. Images were taken using Zeiss 710 Laser Scanning Confocal Microscope. BetaLox5 image was taken with 40X dry objective with a 2.5X digital zoom. T cell images were taken with a 63X oil objective with a 3X digital zoom, 3D image of T cells was processed as video (Supplemental video) and as maximum intensity projection (Fig. 1A). Colocalization parameters were calculated using Zeiss Zen2.1 software (Carl Zeiss).

### Statistics

Data were analyzed using GraphPad Prism (Version 6.0g, GraphPad Software, Inc) and JMP 7 (SAS Institute Inc). One-way ANOVA with Bonferroni post-hoc corrections was used for multiple comparisons, and unpaired t-test with Welch’s correction was used when comparing two groups. Correlations were conducted by Spearman analysis or Multivariate correlation analysis. For paired samples, stimulated and isotype control treated conditions were compared via Wilcoxon test. Non-parametric analyses were conducted by Mann-Whitney tests with p < 0.05 deemed significant.

### Data availability

The datasets generated during and/or analyzed during the current study are available from the corresponding author on reasonable request.

### Prior Presentations

Parts of this study were presented in abstract form at 12^th^ Annual Meeting of the Federation of Clinical Immunology Societies, Vancouver, Canada, June 2012, and 73^rd^ American Diabetes Association Scientific Session, Chicago, June 2013, Immunology of Diabetes Society 14^th^ International Congress, Munich, Germany, April 2015, and American Diabetes Association Scientific Session, New Orleans, June 2016.

## Electronic supplementary material

Supplementary Information

Supplemental video
