# Supplementary material for: T cells display mitochondria hyperpolarization in human type 1 diabetes
Source: Sci Rep. 2017 Sep 7;7:10835. doi: 10.1038/s41598-017-11056-9 (PMC5589742; doi:10.1038/s41598-017-11056-9)
Supplement: Supplementary file 1 — Supplementary Information [file 41598_2017_11056_MOESM1_ESM.doc]

**T cells display mitochondria hyperpolarization in human type 1 diabetes**

Running Title: T cell MHP in type 1 diabetes

Jing Chen^1*^, Anna V. Chernatynskaya^1^, Jian-Wei Li^1,2^, Matthew R. Kimbrell^1^, Richard J. Cassidy^1,3^, Daniel J. Perry^1^, Andrew B. Muir^4^, Mark A. Atkinson^1^, Todd M. Brusko^1^, Clayton E. Mathews^1^

1, Department of Pathology, Immunology, and Laboratory Medicine, University of Florida, Gainesville, FL

2, Present affiliation: Department of Endocrinology and Metabolism, West China Hospital of Sichuan University, Chengdu, China 610041

3, Present affiliation: Department of Radiation Oncology, Emory University, Atlanta, GA

4, Department of Pediatrics, Emory University, Atlanta, GA

*Corresponding author: Dr. Jing Chen, Department of Pathology, Immunology, and Laboratory Medicine, The University of Florida College of Medicine, 1275 Center Drive, P.O. Box 100275

Gainesville, FL 32610-0275, 352-273-9262, Fax: 352-273-9339, e-mail: [jic24@pathology.ufl.edu](mailto:jic24@pathology.ufl.edu)

Table S1. Pairwise correlation analysis of nine T cell subsets for ΔΨm

| **Variable 1** | **Variable 2** | **Correlation** | **Count** | **Signif Prob** |
| --- | --- | --- | --- | --- |
| CD4+ | CD3+ | 0.9854 | 107 | <.0001 |
| CD4+ T conv | CD3+ | 0.9827 | 107 | <.0001 |
| CD4+ T conv | CD4+ | 0.9964 | 107 | <.0001 |
| CD4+ Memory | CD3+ | 0.9519 | 107 | <.0001 |
| CD4+ Memory | CD4+ | 0.9705 | 107 | <.0001 |
| CD4+ Memory | CD4+ T conv | 0.9787 | 107 | <.0001 |
| CD4+ Naïve | CD3+ | 0.9720 | 107 | <.0001 |
| CD4+ Naïve | CD4+ | 0.9649 | 107 | <.0001 |
| CD4+ Naïve | CD4+ T conv | 0.9642 | 107 | <.0001 |
| CD4+ Naïve | CD4+ Memory | 0.9306 | 107 | <.0001 |
| Treg | CD3+ | 0.9328 | 107 | <.0001 |
| Treg | CD4+ | 0.9442 | 107 | <.0001 |
| Treg | CD4+ T conv | 0.9248 | 107 | <.0001 |
| Treg | CD4+ Memory | 0.8896 | 107 | <.0001 |
| Treg | CD4+ Naïve | 0.9375 | 107 | <.0001 |
| CD8+ T cells | CD3+ | 0.9770 | 107 | <.0001 |
| CD8+ T cells | CD4+ | 0.9526 | 107 | <.0001 |
| CD8+ T cells | CD4+ T conv | 0.9430 | 107 | <.0001 |
| CD8+ T cells | CD4+ Memory | 0.9118 | 107 | <.0001 |
| CD8+ T cells | CD4+ Naïve | 0.9388 | 107 | <.0001 |
| CD8+ T cells | Treg | 0.9212 | 107 | <.0001 |
| CD8+ Memory | CD3+ | 0.7988 | 107 | <.0001 |
| CD8+ Memory | CD4+ | 0.7889 | 107 | <.0001 |
| CD8+ Memory | CD4+ T conv | 0.7715 | 107 | <.0001 |
| CD8+ Memory | CD4+ Memory | 0.7620 | 107 | <.0001 |
| CD8+ Memory | CD4+ Naïve | 0.7612 | 107 | <.0001 |
| CD8+ Memory | Treg | 0.7331 | 107 | <.0001 |
| CD8+ Memory | CD8+ T cells | 0.8298 | 107 | <.0001 |
| CD8+ Naïve | CD3+ | 0.9379 | 107 | <.0001 |
| CD8+ Naïve | CD4+ | 0.9265 | 107 | <.0001 |
| CD8+ Naïve | CD4+ T conv | 0.9155 | 107 | <.0001 |
| CD8+ Naïve | CD4+ Memory | 0.8935 | 107 | <.0001 |
| CD8+ Naïve | CD4+ Naïve | 0.9374 | 107 | <.0001 |
| CD8+ Naïve | Treg | 0.9292 | 107 | <.0001 |
| CD8+ Naïve | CD8+ T cells | 0.9416 | 107 | <.0001 |
| CD8+ Naïve | CD8+ Memory | 0.6762 | 107 | <.0001 |

Table S2. Colocalization of DiOC6 and Mitotracker Deep Red (MTDR)

| **Parameters** | **value** |
| --- | --- |
| Co-localization Coefficient Ch2 M (MTDR) | 0.949 |
| Co-localization Coefficient Ch3 (DiOC6) | 0.873 |
| Weighted Co-localization Coefficient Ch2 M (MTDR) | 0.973 |
| Weighted Co-localization Coefficient Ch2 M (MTDR) | 0.926 |
| Overlap Coefficient | 0.91 |
| Correlation R | 0.68 |
| Correlation RXR | 0.46 |

**Supplemental Figure Legends**

**Figure S1**. Low dose DiOC6 is restricted in mitochondria. Human Beta cell line betaLox5 was stained with Hoechst 33258 (2 µg/mL, Blue), DiOC6 (20nM, green) and Mitotracker Deep Red (10nM, Red). Images were taken with Zeiss 710 laser confocal microscope, 40X objective. Overlay (lower right panel) shows co-localization of low dose DiOC6 and Mitotracker Deep Red.

**Figure S2**. There is no correlation of T cell ΔΨm with duration of T1D (A) or patients’ blood glucose level at sample collection (B).

**Figure S3**. There is no difference in activation-induced Nitric Oxide (NO) production from T cells of T1D patients, first degree relatives or healthy controls.

**Figure S4**. There is no correlation of T cell ΔΨm with glycolysis. (A) A representative extracellular acidification rate (ECAR) record. Glycolysis parameters of enriched T cells were measured using an XF 24 Extracellular Flux Analyzer. Antibodies and respiratory inhibitors were injected at each time point as mentioned in method session. Activation-induced (AI-) changes were calculated as the difference between activating antibody treated value (light blue) and isotype antibody treated value (dark blue). No correlation was detected between ΔΨ and (B) basal glycolysis, AI-ECAR, (C) glycolytic capacity, AI-glycolytic capacity, (D) proton production, AI-proton production. AUC: area under curve.

**Figure S3**

**Figure S4**
